# How Often Do You Think About Your Relationship With Nature? The Measurement of Environmental Identity Salience and Its Relationship With Proenvironmental Behaviors

**DOI:** 10.3389/fpsyg.2022.877978

**Published:** 2022-07-08

**Authors:** Leïla Rahmani, Simona Haasova, Sandor Czellar, Valentina Clergue, Christian Martin

**Affiliations:** ^1^Department of Marketing, Faculty of Business and Economics, University of Lausanne, Lausanne, Switzerland; ^2^EHL Hospitality Business School, HES-SO University of Applied Sciences and Arts of Western Switzerland, Lausanne, Switzerland; ^3^School of Business, Maynooth University, Maynooth, Ireland

**Keywords:** proenvironmental behaviors, environmental identity, identity strength, identity salience, sustainable consumption, measurement development, nature connectedness

## Abstract

Extant research finds that environmental identity is an important motivational factor for proenvironmental behavior. However, studies typically focus on investigating the effects of the strength of this identity. Based on insights from identity research, we theorize that the influence of individuals’ environmental identity on their proenvironmental behavior may depend on other identity dimensions as well. We argue that the frequency of activation of environmental identity in relevant life domains—environmental identity salience—may predict proenvironmental behavior beyond what environmental identity strength can explain. To test our theorizing, we propose a parsimonious measure of environmental identity salience. In four empirical studies, we establish that the new measure has sound psychometric properties in terms of internal consistency and discriminant validity with regard to measures of environmental identity strength. Importantly, our measure of environmental identity salience reliably predicts a range of self-reported and actual proenvironmental behaviors beyond the effects of environmental identity strength. In line with theoretical predictions, our data suggests that environmental identity salience and strength are related but distinct constructs. We conclude that investigating the nature and effects of environmental identity salience leads to a fruitful path to a more comprehensive understanding of proenvironmental behavior. The proposed new measure may serve as a helpful tool in this endeavor.

## Introduction

Since its advent in the early 2000’s, the concept and relevant measures of environmental identity have become central elements in research focusing on the psychological processes underlying proenvironmental behavior (e.g., Clayton, 2003, 2012; Pritchard et al., 2020; Lou and Li, 2021; Schultz, 2001). The bulk of this research has focused on one specific aspect of environmental identity, *identity strength*, which refers to the intensity of an individual’s sense of psychological relation between the natural environment and the self. Literature suggests that individuals who report a stronger (weaker) environmental identity are more (less) likely to exhibit proenvironmental attitudes and behavioral intentions, and engage in concrete sustainable behaviors (e.g., Mayer and Frantz, 2004; Tam, 2013; Frantz and Mayer, 2014; Martin and Czellar, 2016). Indeed, the relationship between environmental identity strength and behavioral tendencies has consistently been shown to be positive in meta-analytical reviews (Mackay and Schmitt, 2019; Whitburn et al., 2020; Vesely et al., 2021). Yet, research in identity theory suggests that there might be further explanatory constructs underlying the influence of environmental identity on engagement in proenvironmental behaviors.

In the current research, we present evidence suggesting that the salience of an individual’s environmental identity may predict proenvironmental behavior above and beyond the effect attributable to environmental identity strength when individuals engage in environmentally relevant behaviors. Salience of an identity pertains to the likelihood of its activation in an individual’s mind, which is an important factor for identity-congruent behavioral motivations to occur (Reed, 2004). Nature-protecting motivations, which represent focal drives of identity-based behaviors in a conservational context, are particularly pertinent for shaping behavior in environmentally relevant domains (Schultz and Kaiser, 2012). We thus conceptualize *environmental identity salience* as the frequency of the identity’s activation in behavioral domains of everyday life that are environmentally relevant. We argue that individuals whose environmental identity is more (less) frequently salient in common proenvironmental domains will be more (less) likely to engage in proenvironmental behavior.

Based on this theorizing, the present research introduces a novel perspective on the assessment of environmental identity and provides its first applications in the prediction of various proenvironmental behaviors. In four studies, we show that a proposed new tool for the measurement of environmental identity salience meaningfully and uniquely predicts both self-reported and actual conservational behavior in a variety of domains ranging from product choices to donations to support for environmental policies. Our findings indicate that considering the salience of individual environmental identity, in addition to its strength, may result in more accurate predictions of identity-based proenvironmental behaviors. Our studies also show that strength and salience are related, yet distinct dimensions of environmental identity, each being uniquely associated with behavioral tendencies. Our empirical results suggest that future inquiries into identity-based conservational behavior may benefit from adopting a more comprehensive perspective on environmental identity that goes beyond identity strength and includes considerations related to other identity dimensions as well.

### Conceptual Framework

In recent years, research in environmental psychology has devoted considerable attention to the study of self-definitional mechanisms underlying proenvironmental behavior. For example, the metapersonal self-construal (i.e., the extent to which people see themselves as being interdependent with all living beings on Earth) seems to be positively associated with environmental conservation tendencies (Arnocky et al., 2007). Research also indicates that interpersonal variations in perceived global self-definition, corresponding to a sense of identification with all humans, positively relates to self-reported proenvironmental behavior (Loy and Reese, 2019). Place attachment and its sub-dimension of place identity, the latter being defined as a sense that the self is related to a specific geographical location, are also associated with a more pronounced propensity for proenvironmental behavior (Ramkissoon et al., 2013; Ramkissoon and Mavondo, 2015; Daryanto and Song, 2021).

The self-definitional concept that is particularly prominent in the study of identity-based processes in proenvironmental behavior is environmental identity. One of the most influential and comprehensive definitions of environmental identity refers to it as “a sense of connection to some part of the non-human environment that affects the way we perceive and act toward the world; the belief that the environment is important to us and an important part of who we are” (Clayton, 2003, p. 45–46). Environmental identity is thus conceptualized as a working relationship between the self and the natural environment that can affect the way humans view their surroundings and behave with respect to them. Extant research has proposed a series of measurement tools to gauge environmental identity, including versions of the Environmental Identity Scale (Clayton, 2003, Clayton et al., 2021), versions of the Inclusion of Nature in Self Scale (Schultz, 2001; Martin and Czellar, 2016), the Connectedness to Nature Scale (Mayer and Frantz, 2004), versions of the Nature Relatedness Scale (Nisbet et al., 2009; Nisbet and Zelenski, 2013) and the Nature Connection Index (Richardson et al., 2019). A common feature of these psychometric scales is an emphasis on the assessment of the perceived relational strength between humans’ self and the natural environment, a dimension of environmental identity that can be qualified as *identity strength*. Findings regarding the behavioral implications of environmental identity have been summarized in two meta-analyses (Mackay and Schmitt, 2019; Whitburn et al., 2020). A third, comprehensive set of meta-analyses by Vesely et al. (2021) included a review of the relationship between connectedness to nature and climate-friendly intentions and self-reported behavior. The results of these reviews indicate a consistently positive, albeit varying in strength and context-dependent, relationship between individual environmental identity and conservational behavioral tendencies.

While the focus on the general strength of individual’s environmental identity has substantially contributed to advancing our knowledge about the importance of humans’ relationships with the natural environment, a more comprehensive stance may help us improve our understanding of the complexity of the linkages between environmental identity and ensuing sustainable behaviors. We propose that, in an effort to better apprehend identity-based individual behavior, research may benefit from a more global approach to the assessment of environmental identity characteristics by measuring other dimensions of environmental identity as well. In the present research, we propose to study the effects of one such dimension—the salience of environmental identity in common proenvironmental domains.

The conceptual backbone of our research is derived from identity theories (Oyserman, 2009; Reed et al., 2012), which posit that a key condition for the enactment of a given identity in a decision context is the activation of that identity in an individual’s mind, defined as the identity’s salience. When an identity is salient, the attitudes and behavioral intentions that are congruent with it are brought to the forefront of an individual’s mind and are more likely to be acted upon (Reed, 2004). If, for instance, an identity such as “athlete” is salient for an individual, that individual might be more likely to heed to information that is relevant to that identity, such as sports news, or consume athletics-related products, such as protein bars (Reed and Forehand, 2016). Importantly, while an identity can be situationally activated through identity-related cues, it can also be more or less chronically salient across behavioral domains (Reed et al., 2012). That is, for some individuals it can be generally on their minds across different areas of decision making, while for some other individuals, the identity may occupy their thoughts less across various domains (Oyserman, 2009). It is therefore theorized that the frequency of activation of an environmental identity in identity-relevant domains increases the probability that the identity will have a subsequent influence on the person’s behavioral tendencies and actual behaviors (Reed, 2004; Reed et al., 2012).

On the basis of the preceding theoretical insights, we propose the construct of *environmental identity salience*, conceptualized as the frequency of activation of an individual’s environmental identity in proenvironmental domains of everyday life. We contend that assessing whether environmental identity is more or less frequently salient, and thus active, for individuals in various decision domains will be an important and valuable predictor of proenvironmental behaviors. Such a measure would indicate to what extent an individuals’ connection to nature is factored into individual decisions in various fields of environmentally relevant behaviors (e.g., consumption, transport, or waste disposal). However, in our reading, the commonly used scales, referred to above, do not directly capture the salience dimension of environmental identity.

We thus argue that environmental identity salience could be a meaningful and complementary identity aspect that can help us better understand, and predict, identity-based proenvironmental behaviors. In line with previous research (e.g., Mackay and Schmitt, 2019; Whitburn et al., 2020; Vesely et al., 2021), we expect that individuals will be more motivated to engage in proenvironmental actions if their environmental identity is stronger. Theory suggests that environmental identity salience and strength are related yet distinct constructs in their functions and effects (Stryker and Serpe, 1994; Reed, 2004; Reed et al., 2012). We thus predict that for individuals with similarly strong environmental identities, those with more salient identities should engage more in proenvironmental behavior than those who experience lower levels of environmental identity salience.

Therefore, in an attempt to predict proenvironmental behavior on the grounds of identity-based measures, it could be worthwhile to include not only individuals’ baseline environmental identity strength, but also the frequency with which environmental identity is salient in domains that are most relevant to environmental protection. The main hypothesis of our research is that by considering the salience of environmental identity, it may be possible to predict individual variations in proenvironmental behavioral tendencies over and above the effects attributable to the general strength of environmental identity alone.

### Overview of Studies

We test this hypothesis in four empirical studies by distinguishing between two particular aspects of environmental identity—*environmental identity strength* and *environmental identity salience.* We first asses the dimensionality, internal consistency and discriminant validity of the proposed environmental identity salience measure, and then examine the predictive power of the environmental identity strength and salience measures using a series of self-reported and actual proenvironmental behaviors.

To assess environmental identity salience, we propose a new measure based on definitions of identity salience by Reed et al. (2012) and Kettle (2019), assessing the extent to which environmental identity occupies one’s thoughts in common proenvironmental domains. We do so by measuring how frequently individuals think of their environmental identity in different broad environmentally-relevant domains of behavior based on Schultz and Kaiser (2012).

Studies 1 and 1b represent a preliminary, cross-sectional investigation of our hypothesis about the distinct effects of environmental identity strength and salience on the self-reported enactment of proenvironmental behaviors. Data from studies 1 and 1b are also used for in-depth analyses of the salience measure in regard to its internal consistency, dimensionality and its relationship with measures of environmental identity strength. Study 2 tests our theorizing with observed, actual proenvironmental behaviors in a controlled laboratory setting. Study 3 examines the effects of environmental identity strength and salience in a longitudinal study using a nationally representative sample of citizens. Compared to the previous studies, we test our hypothesis with yet another type of proenvironmental action—public voting on support/rejection of environmental policy implementation for global corporations. The longitudinal nature of the data also allows us to assess the temporal stability of the proposed new tool for the assessment of environmental identity salience.

The exhaustive list of the relevant measures, their sources, concrete items and response formats, including scale reliability statistics and descriptive statistics for each study in this paper can be found in Supplementary Material. The datasets can be found here: https://drive.switch.ch/index.php/s/oUkl0sQ4VnFhc8p.

## Study 1

Study 1 is a preliminary investigation of our prediction about the effects of environmental identity strength and salience with regard to the enactment of a series of proenvironmental behaviors.

### Participants

We conducted an online survey on Amazon Mechanical Turk with 502 participants (*M*_age_ = 36.48, 53% male) in exchange for a standard payment. We removed 25 participants (4.98% of the initial sample) because they failed an embedded attention check or did not complete the survey entirely, which resulted in a final sample of 477 participants for data analysis.

### Design and Procedure

We measured the strength of participants’ environmental identity with the four-item seven-point Extended Inclusion of Nature in Self (EINS) scale (*M* = 4.88, *SD* = 1.19, α = 0.87; Martin and Czellar, 2016; for details, see Supplementary Table B1). Environmental identity salience was measured with our newly developed four-item seven-point scale (*M* = 4.83, *SD* = 1.27, α = 0.80; for details, see Supplementary Table B1). This measure assesses salience of an identity as a function of the frequency with which it is considered and occupies one’s thoughts in various situations. Specifically, participants were asked the question: “In the following aspects of your daily life, how often do you think about your relationship with the natural environment?” They reported answers on a four-item, seven-point scale anchored with “never” and “very often” that included four broad domains relevant to proenvironmental behavior: House-related activities, activities related to transportation and traveling, activities related to waste disposal and consumption-related activities. These categories were created based on the classification of proenvironmental action domains proposed by Schultz and Kaiser (2012).

Engagement in self-reported proenvironmental behaviors was measured with a 12-item seven-point scale (*M* = 4.45, *SD* = 1.25, α = 0.91; Tam, 2013; for details, see Supplementary Table B1) assessing how frequently a participant performed various proenvironmental behaviors. Sample items from the scale included statements such as “purchasing products in reusable containers,” “volunteering time to help an environmentalist group,” and “taking a shorter shower to conserve water” (Tam, 2013).

To avoid order effects, we randomized the order of presentation of the three scales and additionally embedded them within a larger set of unrelated measures. An attention check item was incorporated in an unrelated set of questions that was also presented within the random order of the questionnaire. Demographic information appeared at the end of the survey.

### Results and Discussion

We first intended to establish that environmental identity strength and salience indeed represented two related but distinct constructs. The correlation between the measures of the two constructs was positive (*r* = 0.56, *p* < 0.001). We used exploratory factor analysis (EFA) for the set of eight items composing the two scales. The Kaiser–Meyer–Olkin value was 0.87, which is above the recommended threshold of 0.6 (Kaiser, 1974), and Bartlett’s Test of sphericity achieved statistical significance (*p* < 0.001), indicating that the correlations were large enough for EFA. Two factors explaining 67.38% of the variance in the data were extracted. We decided on the number of factors from the eigenvalues, cumulative variance, and inspection of the scree plot. We rotated the factors obliquely using Promax rotation (correlated data); interpretation of the two factors was in line with our two-dimensional conceptualization of environmental identity (i.e., strength and salience). Each item loaded on its expected respective factor (for details, see Table 1).

**TABLE 1:** Summary of EFA results (Study 1).

	Factor loadings	
Item	1	2
Overlap^a^	**0.77**	0.53
Size^a^	**0.69**	0.47
Distance^a^	**0.86**	0.52
Central^a^	**0.83**	0.54
House-related activities^b^	0.45	**0.65**
Activities related to transportation and traveling^b^	0.51	**0.73**
Activities related to waste disposal^b^	0.42	**0.64**
Consumption-related activities^b^	0.52	**0.83**

*Instructions preceding each item: ^a^“Below, please choose the pictures which best describe your relationship with the natural environment”; ^b^“In the following aspects of your daily life, how often do you think about your relationship with the natural environment.” The bold values represent dominant scale item loadings onto each of the factors.*

We next regressed engagement in proenvironmental behaviors on the mean-centered environmental identity strength and salience measures separately (model 1 and model 2) and jointly (model 3) (for an overview of the statistical results, see Table 2). We found effects for both, environmental identity strength and salience as single predictors, with salience seemingly having a more pronounced main effect. When both were entered into the model simultaneously, the main effects of strength and salience became weaker but remained statistically significant. Salience predicted engagement in proenvironmental behaviors more strongly compared to strength in that model.

**TABLE 2:** Linear regression models (Study 1).

		Dependent variable	
		Engagement in proenvironmental behaviors	
Model	Predictors	Coefficients	Model statistics
Model 1	*EI strength*	β = 0.59, *t* = 15.84, *p* < 0.001	*F*_(1,475)_ = 250.85, *p* < 0.001, _adj_*R*^2^ = 0.34
Model 2	*EI salience*	β = 0.73, *t* = 23.19, *p* < 0.001	*F*_(1,475)_ = 537.54, *p* < 0.001, _adj_*R*^2^ = 0.53
Model 3	*EI strength* ^a^	β = 0.26, *t* = 7.13, *p* < 0.001	*F*_(2,474)_ = 322.42, *p* < 0.001, _adj_*R*^2^ = 0.58
	*EI salience*	β = 0.58, *t* = 16.07, *p* < 0.001	

*EI, Environmental identity. The effects are in standardized beta coefficients. ^a^VIF = 1.47.*

In a follow-up Study 1b, we aimed to replicate these results using the Revised Environmental Identity scale (Clayton et al., 2021) instead of the Extended Inclusion of Nature in Self scale (Martin and Czellar, 2016). The results of Study 1b corroborate Study 1 with a different measure of environmental identity strength. This suggests that our findings from Study 1 are not specific to a particular operationalization of environmental identity strength (for detailed statistics, see “Study 1b” in Supplementary Material).

Overall, Studies 1 and 1b provide preliminary evidence for the relation of environmental identity salience, in addition to strength, with engagement in proenvironmental behaviors. This evidence should be considered as preliminary due to methodological limitations, which include in particular: (1) the self-reports of engagement in proenvironmental behavior and (2) the concurrent measurement of the independent and dependent variables in a (randomized) sequence. Both of these concerns may have inflated our correlational results. To strengthen the validity and generalizability of our findings, we address these limitations in the next two studies.

## Study 2

The purpose of Study 2 was to further examine the predictive effects of environmental identity strength and salience on actual, rather than self-reported, proenvironmental behaviors in consumer product choices. Previous research indicates that consumers often use products to enact their identities. Thus, product choices provide a relevant and meaningful context for the investigation of identity-based effects in individual behavior (Kleine et al., 1993).

### Participants

We conducted a laboratory study with 391 participants (*M*_age_ = 21.16, 47% male) in exchange for a standard payment. We removed three participants (0.8%) who had failed an embedded attention check, which left a final sample of 388 participants for our main analyses.

### Design and Procedure

The study comprised two parts. In the first part (embedded among other unrelated materials) participants were presented with two choice tasks and, for each of those, were instructed to choose one of two product alternatives: apple (organic vs. conventionally grown) and regular Coke (in a glass vs. a plastic bottle). The choice task was framed as a choice of an additional reward for study participation that the participants could take with them and consume after the end of the study. The product pairs were placed in front of the participants on a desk in their laboratory cubicle. A small commercial “Bio” label was affixed to the organic apple, while the conventionally grown apple was unmarked. Both Coke alternatives used their original packaging. Choice of an organic (vs. conventional) apple and glass (vs. plastic) bottle of Coke corresponded to more (vs. less) proenvironmental behaviors. To create a tradeoff between choosing the more, or the less, proenvironmental product alternative, the organic apples were smaller than the conventional apples, though the variety (Golden) was the same in both conditions. The glass bottle was also smaller (250 ml) than the plastic bottle (450 ml) of the Coke. After that, we also measured participants’ willingness to donate to a proenvironmental organization, the World Wide Fund for Nature (WWF). To do so, we told participants that they would enter a raffle after the experiment in which two participants would be drawn to win the equivalent of US $100. We then asked them how much of that money they would be willing to donate to the WWF should they be one of the winners (we eventually indeed donated the amount the two winners had specified, and they received the difference between their prize and the donated amount). In the second part of the study, participants completed the environmental identity strength (*M* = 4.72, *SD* = 1.02, α = 0.86) and salience (*M* = 5.04, *SD* = 1.14, α = 0.67) measures, which were the same as those in Study 1. These measures were randomized and embedded in a larger set of unrelated measures that also included an attention check item. Demographic questions appeared at the end.

### Results and Discussion

In line with the results of Study 1, we found a positive correlation between the two characteristics of environmental identity: strength and salience (*r* = 0.56, *p* < 0.001).

Next, we performed a series of logistic regressions by separately regressing each of the choice measures on the mean-centered environmental identity strength and salience measures in models with the two measures as separate predictors (model 1 and model 2) and also entered simultaneously (model 3) (for an overview of the statistical results, see Tables 3, 4). We found separate and similarly strong main effects for both, environmental identity strength and salience, on more sustainable product choice of Coke (glass bottle) and apple (organic). When both predictors were entered into the model simultaneously, the strength and salience effects remained both statistically significant in the case of apple choice, but the strength main effect became statistically non-significant in the choice of the Coke bottle.

**TABLE 3:** Logistic regression models (Study 2).

		Dependent variable			
		Coca-Cola bottle choice^a^		Apple choice^a^	
Model	Predictors	Coefficients	Model statistics	Coefficients	Model statistics
Model 1	*EI strength*	β = 0.31, χ^2^(1) = 8.85, *p* = 0.003	χ^2^(1) = 9.17, *p* < 0.001, Nagelkerke *R*^2^ = 0.03	β = 0.70, χ^2^(1) = 34.88, *p* < 0.001	χ*^2^*(1) = 40.73, *p* < 0.001, Nagelkerke *R*^2^ = 0.14
Model 2	*EI salience*	β = 0.33, χ^2^(1) = 12.29, *p* < 0.001	χ^2^(1) = 12.87, *p* < 0.001, Nagelkerke *R*^2^ = 0.04	β = 0.63, χ^2^(1) = 35.43, *p* < 0.001	χ*^2^*(1) = 41.08, *p* < 0.001, Nagelkerke *R*^2^ = 0.14
Model 3	*EI strength*	β = 0.15, χ^2^(1) = 1.55, *p* = 0.214	χ^2^(2) = 14.42, *p* < 0.001, Nagelkerke *R*^2^ = 0.05	β = 0.46, χ^2^(1) = 12.05, *p* < 0.001	χ*^2^*(2) = 53.21, *p* < 0.001, Nagelkerke *R*^2^ = 0.17
	*EI salience*	β = 0.25, χ^2^(1) = 5.17, *p* = 0.023		β = 0.42, χ^2^(1) = 11.56, *p* < 0.001	

*EI, Environmental identity. ^a^ 0 = less sustainable / 1 = more sustainable.*

**TABLE 4:** Linear regression models (Study 2).

		Dependent variable	
		*Donation to WWF*	
Model	Predictors	Coefficients	Model statistics
Model 1	*EI strength*	β = 0.11, *t* = 2.17, *p* = 0.030	*F*_(1,386)_ = 4.72, *p* = .03, _adj_*R*^2^ = 0.01
Model 2	*EI salience*	β = 0.15, *t* = 3.00, *p* = 0.003	*F*_(1,386)_ = 8.99, *p* = .003, _adj_*R*^2^ = 0.02
Model 3	*EI strength^a^*	β = 0.04, *t* = 0.61, *p* = 0.543	*F*_(2,385)_ = 4.67, *p* = .01, _adj_*R*^2^ = 0.02
	*EI salience*	β = 0.13, *t* = 2.14, *p* = 0.033	

*EI, Environmental identity.*

*^a^VIF = 1.46.*

Furthermore, linear regressions revealed a significant main effect of environmental identity strength and environmental identity salience on the amount participants were willing to donate to the WWF, when entered as single predictors. When both measures were entered into the model, we found a main effect of salience but no longer a main effect of environmental identity strength. The models including environmental identity salience explained more variance in the data than the model without it.

Using real product choices and donation behavior, this study corroborates our initial findings and provides additional support for the potential value of our measure of individuals’ environmental identity salience with respect to predicting engagement in proenvironmental behaviors. We also found evidence for a relationship between environmental identity strength and engagement in proenvironmental consumption behaviors, in line with the empirical literature (e.g., Mackay and Schmitt, 2019). Importantly, our results indicate that environmental identity salience had a consistent association with a variety of proenvironmental choices and behaviors.

It appears from our findings that environmental identity salience predicts individuals’ proenvironmental actions above and beyond environmental identity strength (and in some cases even more reliably). However, there is an alternative possibility—because in Study 2 participants first made their product choices, reported their donation amount and subsequently answered questions pertaining to their environmental identity strength and salience, the proenvironmental behavioral enactments could have made some participants’ environmental identities temporarily more salient during the study. It could be that our environmental identity salience measure was more sensitive to this effect than the identity strength measure. In order to control for this alternative explanation, the next study tests our predictions using a longitudinal setup.

## Study 3

The purpose of Study 3 was to test our hypotheses in a setting with clear and prolonged temporal distance between the measurement of environmental identity strength/salience and real proenvironmental action—voting behavior in a nation-wide referendum attempting to pass a law ascribing, among others, obligations and responsibilities for environmental protection to multinational businesses (i.e., the Responsible Business Initiative referendum in Switzerland). In addition, we wanted to check if we could corroborate our previous results regarding the significant relations of environmental identity strength and salience to proenvironmental behaviors in a real-life longitudinal context. Lastly, we also intended to test our predictions using another alternative conceptualization and measure of environmental identity strength—environmental self-identity (i.e., personal self-definition as a proenvironmentally acting person; Vesely et al., 2021).

### Participants

Data for this study were collected as part of two waves of a multiple-wave longitudinal study of the general population investigating citizens’ environmental attitudes and behaviors, voting behavior and the impact of the voting outcome in the Responsible Business Initiative referendum that took place on the 29th of November 2020 in Switzerland. Participants for the survey were recruited by a commercial marketing research company that ensured the representativeness of the data in terms of gender, age, and geographical location. Only those who passed an attention check implemented at the very beginning of the survey and fit the available quota combinations (gender, age, region) could complete the survey.

In the first wave, a total of 1101 Swiss residents participated in the survey in exchange for a standard payment between November 20 and 27, 2020. Data for the second wave were collected between December 14 and 23, 2020, approximately 2–4 weeks after the first wave of data collection. A total of 794 participants completed both waves, out of which 535 reported having participated in the voting and 527 also reported how they had voted in the referendum. The demographic characteristics of this sample are as follows: 44.4% women, 53.7% men, 0.4% non-binary (1.9% no response), with a mean age of 50.76 years (*SD* = 16.59, min = 18, max = 86; 3 no response). The formal level of education was university degree for 38.7% of the respondents, high school and similar for 53.7%, and lower than high school and similar for 7.2% (no response: 0.4%).

### Design and Procedure

The study’s design was longitudinal and we measured environmental identity strength, environmental identity salience and self-reported engagement in proenvironmental behaviors across all data collection waves, among other questions. The basic measures of environmental identity strength and salience were identical to studies 1 and 2, but we also measured environmental identity strength with an environmental self-identity measure (the Green Consumer Self-Identity scale, two items, Sparks and Shepherd, 1992, for details, see Supplementary Material). In the presented analyses, we use the measurements of participants’ environmental identity strength (Extended Inclusion of Nature in Self: *M* = 5.14, *SD* = 1.06, α = 0.86; Green Consumer Self-Identity: *M* = 4.90, *SD* = 1.24, α = 0.83) and salience (*M* = 5.08, *SD* = 1.23, α = 0.84) from wave 1 (before the actual referendum) as predictors of participants’ voting behavior in the referendum and engagement in proenvironmental behaviors measured in wave 2 (administered several weeks later and after the actual referendum). We assessed whether citizens reported having voted in the referendum to support or reject the proposed law. Personal engagement in proenvironmental behaviors was measured with the same scale as in Study 1 (12 items; Tam, 2013), enriched by additional 13 items assessing individual performance on a large variety of sustainable consumption behaviors (*M* = 4.20, *SD* = 0.91, α = 0.90). The final score is an average of all the 25 items. In addition, we have also enquired about behaviors related to Christmas shopping and gift-giving (only measured in wave 2)—we asked whether participants engage in seven clearly sustainable types of behavior such as “buying gifts from recycled sources,” “turning off tree lights and indoor/outdoor house decorative lighting at bedtime,” or “reusing gift packing materials,” with response options 0 (does not apply) or 1 (does apply). This measure was self-developed. The final score on this measure was a sum of Christmas-related sustainable behaviors participants reported to engage in during the 2020 Christmas season.

### Results and Discussion

We performed a logistic regression by regressing participants’ vote in the referendum on the Responsible Business Initiative (*N* = 527; in favor = 302, against = 225) assessed in wave 2 on the mean-centered measures of environmental identity strength (using the Extended Inclusion of Nature in Self scale) and salience in models with the two measures as separate predictors (model 1 and model 2) and also entered simultaneously (model 3) (for an overview of the statistical results, see Table 5). The separate analyses revealed statistically significant effects for both environmental identity strength and salience. When both were entered into the model simultaneously, the effect of identity strength became non-significant, and only salience predicted the likelihood of voting in favor of the proenvironmental initiative.

**TABLE 5:** Logistic regression models (Study 3).

		Dependent variable	
		Vote in support of proenvironmental policy (0 = against/1 = in favor)	
Model	Predictors	Coefficients	Model statistics
Model 1	*EI strength*	β = 0.17, χ^2^(1) = 4.20, *p* = 0.040	χ^2^(1) = 4.24, *p* = 0.04, Nagelkerke *R*^2^ = 0.01
Model 2	*EI salience*	β = 0.31, χ^2^(1) = 16.89, *p* < 0.001	χ^2^(1) = 17.72, *p* < 0.001, Nagelkerke *R*^2^ = 0.04
Model 3	*EI strength*	β = −0.04, χ^2^(1) = 0.186, *p* = 0.666	χ^2^(2) = 17.91, *p* < 0.001, Nagelkerke *R*^2^ = 0.05
	*EI salience*	β = 0.34, χ^2^(1) = 13.03, *p* < 0.001	

*EI, Environmental identity.*

The same logistic regression with the measure of Green Consumer Self-Identity instead of the Extended Inclusion of Nature in Self measure for environmental identity strength revealed similar results (see Table 6).

**TABLE 6:** Logistic regression models (Study 3).

		Dependent variable	
		Vote in support of proenvironmental policy (0 = against/1 = in favor)	
Model	Predictors	Coefficients	Model statistics
Model 1	*Green consumer self-identity strength*	β = 0.28, χ^2^(1) = 14.88, *p* < 0.001	χ^2^(1) = 15.40, *p* < 0.001, Nagelkerke *R*^2^ = 0.04
Model 2	*EI salience*	β = 0.31, χ^2^(1) = 16.89, *p* < 0.001	χ^2^(1) = 17.72, *p* < 0.001, Nagelkerke *R*^2^ = 0.04
Model 3	*Green consumer self-identity strength*	β = 0.13, χ^2^(1) = 1.44, *p* = 0.231	χ^2^(2) = 19.15, *p* < 0.001, Nagelkerke *R*^2^ = 0.05
	*EI salience*	β = 0.22, χ^2^(1) = 3.70, *p* = 0.054	

*EI, Environmental identity.*

We have also performed linear regression analyses, using self-reported engagement in proenvironmental behaviors (measured in wave 2, *N* = 794) and engagement in sustainable Christmas shopping and gift-giving behaviors (measured in wave 2, *N* = 606) as dependent variables (see Table 7). We again found significant effects for environmental identity strength and salience.

**TABLE 7:** Linear regression models (Study 3).

		Dependent variable			
		Engagement in proenvironmental behaviors		Engagement in sustainable Christmas behaviors	
Model	Predictors	Coefficients	Model statistics	Coefficients	Model statistics
Model 1	*EI strength*	β = 0.46, *t* = 14.66, *p* < 0.001	*F*_(1, 792)_ = 214.99, *p* < 0.001, _adj_*R*^2^ = 0.21	β = 0.18, *t* = 4.38, *p* < 0.001	*F*_(1,604)_ = 31.49, *p* < 0.001, _adj_*R*^2^ = 0.03
Model 2	*EI salience*	β = 0.67, *t* = 25.20, *p* < 0.001	*F*_(1,792)_ = 634.78, *p* < 0.001, _adj_*R*^2^ = 0.44	β = 0.32, *t* = 8.27, *p* < 0.001	*F*_(1,604)_ = 68.41, *p* < 0.001, _adj_*R*^2^ = 0.10
Model 3	*EI strength^a^*	β = 0.13, *t* = 4.17, *p* < 0.001	*F*_(2,791)_ = 332.68, *p* < 0.001, _adj_*R*^2^ = 0.46	β = −0.003, *t* = −0.06, *p* = 0.96	*F*_(2,603)_ = 34.15, *p* < 0.001, _adj_*R*^2^ = 0.10
	*EI salience*	β = 0.59, *t* = 18.83, *p* < 0.001		β = 0.32, *t* = 6.90, *p* < 0.001	

*EI, Environmental identity. The effects are in standardized beta coefficients. ^a^VIF = 1.45/1.45.*

Similar results were obtained with the green consumer self-identity as a measure of environmental identity strength (see Table 8).

**TABLE 8:** Linear regression models (Study 3).

		Dependent variable			
		Engagement in proenvironmental behaviors		Engagement in sustainable Christmas behaviors	
Model	Predictors	Coefficients	Model statistics	Coefficients	Model statistics
Model 1	*Green consumer self-identity strength*	β = 0.67, *t* = 25.04, *p* < 0.001	*F*_(1,792)_ = 626.87, *p* < 0.001, _adj_*R*^2^ = 0.44	β = 0.28, *t* = 7.11, *p* < 0.001	*F*_(1,604)_ = 50.55, *p* < 0.001, _adj_*R*^2^ = 0.08
Model 2	*EI salience*	β = 0.67, *t* = 25.20, *p* < 0.001	*F*_(1,792)_ = 634.78, *p* < 0.001, _adj_*R*^2^ = 0.44	β = 0.32, *t* = 8.27, *p* < 0.001	*F*_(1,604)_ = 68.41, *p* < 0.001, _adj_*R*^2^ = 0.10
Model 3	*Green consumer self-identity strength^a^*	β = 0.38, *t* = 10.19, *p* < 0.001	*F*_(2,791)_ = 410.54, *p* < 0.001, _adj_*R*^2^ = 0.51	β = 0.10, *t* = 1.70, *p* = 0.091	*F*_(2,603)_ = 35.75, *p* < 0.001, _adj_*R*^2^ = 0.10
	*EI salience*	β = 0.39, *t* = 10.43, *p* < 0.001		β = 0.25, *t* = 4.41, *p* < 0.001	

*EI, Environmental identity. The effects are in standardized beta coefficients. ^a^VIF = 2.22/2.15.*

Both of the focal environmental identity characteristics, strength and salience, remained quite stable over the 2–4 week period between waves 1 and 2 of data collection [strength wave 1 with wave 2: *r*(794) = 0.71, *p* < 0.001: salience wave 1 with wave 2: *r*(794) = 0.73, *p* < 0.001] and the scores remained quite consistent across time (*M*_*salience*_ wave 1 = 5.08, *SD* = 1.23; *M*_*salience*_ wave 2 = 5.02, *SD* = 1.19; *M*_*strength*_ wave 1 = 5.14 *SD* = 1.06; *M*_*strength*_ wave 2 = 5.06, *SD* = 1.13). The two constructs were positively correlated at both times [strength_salience wave 1: *r*(794) = 0.56, *p* < 0.001; strength_salience wave 2: *r*(794) = 0.55, *p* < 0.001].

The results of this study with a representative sample of Swiss citizens replicated our earlier findings using a longitudinal design and investigating a broad portfolio of proenvironmental behaviors and actions. We found that environmental identity salience positively predicted voting behavior in a referendum on a proenvironmental business initiative. The same pattern of results was found with respect to self-reported engagement in a wide range of daily and Christmas-related sustainable behaviors. In addition, in general, the relationships of environmental identity salience with our behavioral measures were stronger compared to the environmental identity strength—behavior relationships. Results were similar when green consumer self-identity was used as an alternative measure of environmental identity strength.

## General Discussion

In the current research, we introduced the concept and associated concise measure of environmental identity salience and tested its psychometric properties in several studies. Our data suggests that our environmental identity salience measure has good internal consistency. Its four items loaded on one factor and factor analyses indicated that they loaded on a different factor than the items of environmental identity strength measures. Our data therefore supports the theorizing that environmental identity salience and strength are related yet distinct constructs (e.g., Stryker and Serpe, 1994; Reed, 2004; Reed et al., 2012).

Importantly, we found that our new measure of environmental identity salience consistently related to a wide range of self-reported and observed actual proenvironmental behaviors, such as choice between regular and environmentally friendly products, donation to an environmental organization, and voting choices on environmental laws. It also predicted proenvironmental behavior when different extant measures of environmental identity strength were included in the models. Interestingly, effect sizes (i.e., *R*^2^) consistently suggest that environmental identity salience may be more strongly related to proenvironmental behaviors than environmental identity strength. The reported environmental identity strength and salience effects were stable across different types of measures of identity strength (i.e., verbal and pictorial). Lastly, a nationally representative longitudinal study indicated that both environmental identity strength and salience are temporarily stable, at least over the course of a few weeks, and that they predict proenvironmental behavior even several weeks after their measurements took place.

Noteworthy is the point that the measure of environmental identity salience developed herein is an assessment of an identity’s salience in a rather chronic and cumulative manner. Often, research has conceptualized identity salience as a contextual construct, that is, “a temporary state” of activation of a person’s identity (Reed, 2004, p. 286). This contextual perspective often implies situational manipulation of an identity that involves experimental setups and specifically designed stimuli for the focal identity at hand. However, chronic properties have been acknowledged in extant literature as well: “which aspect of identity comes to mind is a dynamic product of that which is chronically accessible and that which is situationally cued” (Oyserman, 2009, p. 250).

### Contribution to Research on Environmental Identity and Behavior

Our research contributes to the literature on environmental identity in several ways. First, we study an identity dimension (i.e., salience) that is established in research on different types of identity, but that appears to be under-researched in relation to environmental identity. Our review of the relevant literature suggested that extant research had focused mostly on the investigation of the strength dimension of environmental identity and how it translates into proenvironmental behavior. We develop a more comprehensive perspective and propose that environmental identity salience can play a meaningful role in proenvironmental behavior in addition to environmental identity strength.

Our findings give a first indication of the merits of such an approach. In particular, our studies consistently indicate that environmental identity salience plays an important role in predicting different types of proenvironmental behavior. However, we do not suggest that environmental identity salience is more important or in any way superior to environmental identity strength. Both concepts play a significant and complementary part in understanding and predicting proenvironmental behavior. Indeed, when predicting proenvironmental behavior in our data, models that included environmental identity salience and strength simultaneously often outperformed models that included only environmental identity strength or only environmental identity salience.

Another contribution of our research lies in the development and validation of a new measurement tool for the salience of environmental identity. We built our measure on the conceptual grounds of identity theory and relevant research in environmental psychology to ensure content relevance and a comprehensive representation of relevant life domains where environmental identity may be salient (Boateng et al., 2018). The application of this measure in empirical studies allowed us to establish its psychometric properties. That is, our measure showed good internal and temporal consistency, and was related to, yet distinct from, the construct of environmental identity strength as measured with different validated scales commonly used in environmental psychology. Our new measure also increased the predictive power of statistical models using various proenvironmental behavioral dependent variables.

The proposed measure may be useful to scholars who wish to study different questions related to environmental identity salience. Because of its parsimonious nature (i.e., only four items), our environmental identity salience measure can be used either as a stand-alone measurement tool or as a complement with scales meant to primarily assess environmental identity strength. Our measure is broad and general in its setup. However, if researchers are interested in studying a particular type of behavior (e.g., energy saving), it is possible to adapt our measure to a specific behavioral context as well.

### Limitations and Future Research

Despite the above-mentioned contributions, our research is limited in several ways. First, our studies do not allow us to explore the relationship between environmental identity strength and salience in detail. On the one hand, our finding of the consistently strong and positive correlation between identity salience and identity strength is in line with the theory-derived assumption that identity salience and identity strength are conceptually related although distinct constructs (Stryker and Serpe, 1994; Reed, 2004; Reed et al., 2012). On the other hand, we cannot draw conclusions as to whether a stronger environmental identity is more likely to be salient more frequently, or whether environmental identity strength may be a consequence of identity reinforcement processes (Reed and Forehand, 2016). Indeed, environmental identity may be strengthened over time through its repeated activation. Apart from providing evidence for concomitant variation between the relevant measures, our data does not allow us to investigate these possible mechanisms directly. Future research could attempt to better understand the relationship and potential feedback loops between the two environmental identity dimensions. Since both dimensions appear to be relatively stable, at least over several weeks (see our Study 3), a longitudinal design that uses a longer timeframe could be employed in such research.

Environmental identity salience and strength might also be related to yet another identity dimension, referred to as *centrality* or *prominence—*the relative standing of a particular identity among other identities within the hierarchy of the self (Stryker and Serpe, 1994; Reed and Forehand, 2016; Kettle, 2019). Identities that are more central to the self are likely to be stronger and more systematically salient and therefore can exert a stronger influence on behaviors across more contexts (Burke, 2006). Our data does again not allow us to investigate this. Future research is needed to shed more light on the structural relationships between environmental identity centrality, salience, and strength.

Next, the items of our environmental identity salience measure are derived from theory and are designed so that they broadly cover the major categories of environmentally relevant behaviors (derived from Schultz and Kaiser, 2012). Nonetheless, our new measurement tool may potentially be limited due to its setup. It may be possible that it does not capture environmental identity salience in general but only in the contexts that are referenced in the individual items. Our data suggests that this is likely not the case. The factor loadings and internal consistency measures indicate that our environmental identity salience measure may tap a broader latent concept. That is, all four items load on one factor highly and items seem to vary together and not independently of one another. Moreover, the measure predicted behaviors that are not within the life domains which the items are based on (e.g., voting behavior). Future research could investigate whether there is benefit in adding additional items to our measure to make it more comprehensive.

Lastly, due to the scope of our research, it was not possible to investigate the wider nomological network around environmental identity salience. Our studies did not focus on individual and situational characteristics that might relate to environmental identity salience. For example, different socio-demographic characteristics (e.g., age, gender, education) may influence environmental identity salience. Similarly, characteristics of an individual’s professional (e.g., type of job) or living (e.g., access to green spaces) environment may also cause individuals to experience environmental identity salience more or less frequently. Future research could study the profiles of high vs. low environmental identity salience individuals to gain a better understanding in this regard. Furthermore, it is important to understand which activities might be more or less likely to enhance such environmental identity activation. For example, literature on eudaemonic identity theory (e.g., Waterman, 2004) and recent findings about perceived flow (Bonaiuto et al., 2016) suggest that enjoyable and optimal flow experiences resulting from self-defining (environmental) activities might be important for the activation of one’s (environmental) identity across situations. This would not only help advance our understanding of identity-based conservational behavior but would also represent valuable knowledge for the development of proenvironmental persuasive messages and a more precise targeting of relevant educational and marketing campaigns.

## Conclusion

Our research suggests that individuals may not only vary in terms of the strength of their relationship with nature, but also in terms of how salient their relationship with nature is to them. Indeed, in our analyses, each environmental identity dimension was associated with unique variation in proenvironmental behavior. A general message of our findings is the need to shift research attention from the study of environment identity strength to a more comprehensive study of different environmental identity dimensions. While environmental identity strength appears to be well researched, much is to be learned about environmental identity salience. Thus, it is not clear when and how individuals develop environmental identity salience, how generalizable it is across proenvironmental behaviors and how variations in salience may be related to individuals’ other identities and their dimensions. For example, heightened environmental identity salience may not just encourage more frequent proenvironmental behavior but may also be in conflict with other goals that individuals may have (Hurst et al., 2013).

Research also suggests that disruptive global events, such as a pandemic through spatial confinement, can facilitate the formation of new types of repeated proenvironmental behaviors in people’s close surroundings (Ramkissoon, 2020). Such developments arguably present opportunities for identity strength and salience effects to emerge in previously under-represented behavioral domains and this at a potentially global scale. Accordingly, the study of such events deserves further attention in the environmental identity literature.

We are confident that zooming in on the idiosyncrasies of various dimensions of individual environmental identities, their relationship with personal motivations and traits, and their intricate effects on ensuing behaviors in conservational domains opens up exciting new areas in environmental psychology and we encourage research that advances our knowledge in this regard.

## Data Availability Statement

The links to the data files in the online repository can be found in the Overview of Studies section.

## Ethics Statement

The studies were reviewed and approved by the Ethics Committee of the Faculty of Business and Economics, University of Lausanne. The participants provided their written informed consent to participate in the studies.

## Author Contributions

LR, SH, and SC designed and conducted the studies together. LR and SH performed the statistical analyses and wrote reports of the study results. SH and SC wrote the first draft of the manuscript. All authors contributed to writing sections of the manuscript and contributed to manuscript revision, read, and approved the submitted version.

## Conflict of Interest

The authors declare that the research was conducted in the absence of any commercial or financial relationships that could be construed as a potential conflict of interest.

## Publisher’s Note

All claims expressed in this article are solely those of the authors and do not necessarily represent those of their affiliated organizations, or those of the publisher, the editors and the reviewers. Any product that may be evaluated in this article, or claim that may be made by its manufacturer, is not guaranteed or endorsed by the publisher.
