# Supplementary material for: How Often Do You Think About Your Relationship With Nature? The Measurement of Environmental Identity Salience and Its Relationship With Proenvironmental Behaviors
Source: Front Psychol. 2022 Jul 8;13:877978. doi: 10.3389/fpsyg.2022.877978 (PMC9305846; doi:10.3389/fpsyg.2022.877978)
Supplement: Supplementary file 1 [file Data_Sheet_1.docx]

Supplementary Material

# Study 1 b

Study 1 b is a replication of Study 1 as described in the manuscript, sharing the design, procedure, and measures with one exception – in Study 1 b environmental identity strength is measured with the Revised Environmental Identity scale (Clayton et al., 2021) instead of the Extended Inclusion of Nature in Self scale (Martin & Czellar, 2016).

## Participants

We conducted an online survey on Amazon Mechanical Turk through the CloudResearch platform with 202 participants in exchange for a standard payment. We removed 2 participants (1 % of the initial sample) because they had failed the attention check, which resulted in a final sample of 200 participants for data analysis (*M*_age_ = 38.96; 0.5 % non-binary, 41.5 % female, 58 % male).

## Design and Procedure

The study’s design and procedure were identical with Study 1.

We measured the strength of participants’ environmental identity with the 14-item seven-point Revised Environmental Identity scale (*M* = 5.35, *SD* = 1.16, *α* = .93; Clayton et al., 2021; for details, see Table B.1 in this document) with response options 1 (Not at all true of me) – 4 (Neither true nor untrue) – 7 (Completely true of me). Environmental identity salience was measured with our newly developed four-item seven-point scale (*M* = 4.46, *SD* = 1.52, *α* = .85; see Table B.1 in this document). Engagement in self-reported proenvironmental behaviors was measured with a 12-item seven-point scale (*M* = 4.16, *SD* = 1.29, *α* = .91; Tam, 2013).

## Results & Discussion

The correlation between the measures of environmental identity strength and environmental identity salience was positive (*r* = .50, *p* < .001).

We used exploratory factor analysis (EFA) for the set of 18 items composing the two scales. The Kaiser–Meyer–Olkin value was .92, which is above the recommended threshold of .6 (Kaiser, 1974), and Bartlett’s Test of Sphericity achieved statistical significance (*p* < .001), indicating that the correlations were large enough for EFA. Three factors explaining 60.16% of the variance in the data were extracted, first factor explaining 46,7 % , second 10,85 % and the last 3.6 %. We decided on the number of factors from the eigenvalues, cumulative variance, and inspection of the scree plot. To interpret the factor loadings, we then rotated the factors obliquely (assuming the factors are not independent and correlated) using Promax rotation. Items from the two scales loaded on their expected respective factors (for details, see Table A1) – the items of the Revised Environmental Identity scale loaded on factor 1 and 2, while the new environmental identity salience measure loaded on factor 3.

Table A1. Summary of EFA Results – zero-order correlations between the items and the extracted factors (Study 1b)

|  | Factor loadings | | |
| --- | --- | --- | --- |
| Item | 1 | 2 | 3 |
| I like to spend time outdoors in natural settings (such as woods, mountains, rivers, fields, local parks, lake or beach, or a leafy yard or garden). ^a^ | **.78** | .54 | .28 |
| I think of myself as a part of nature, not separate from it. ^a^ | .61 | **.77** | .52 |
| If I had enough resources such as time or money, I would spend some of them to protect the natural environment. ^a^ | **.64** | .59 | .51 |
| When I am upset or stressed, I can feel better by spending some time outdoors surrounded by nature. a | **.81** | .63 | .40 |
| I feel that I have a lot in common with wild animals. ^a^ | .43 | **.72** | .40 |
| Behaving responsibly toward nature -- living a sustainable lifestyle -- is important to who I am. ^a^ | .71 | **.73** | .66 |
| Learning about the natural world should be part of everyone’s upbringing. ^a^ | **.72** | .47 | .49 |
| If I could choose, I would prefer to live where I can have a view of the natural environment, such as trees or fields. ^a^ | **.58** | .39 | .26 |
| An important part of my life would be missing if I was not able to get outside and enjoy nature from time to time. ^a^ | **.81** | .47 | .42 |
| I think elements of the natural world are more beautiful than any work of art. ^a^ | **.70** | .46 | .34 |
| I feel refreshed when I spend time in nature. ^a^ | **.87** | .56 | .36 |
| I consider myself a steward of our natural resources. ^a^ | .58 | **.78** | .59 |
| I feel comfortable out in nature. ^a^ | **.77** | .47 | .19 |
| I enjoy encountering elements of nature, like trees or grass, even when I am in a city setting. ^a^ | **.79** | .40 | .37 |
| House-related activities^b^ | .23 | .54 | **.69** |
| Activities related to transportation and traveling^b^ | .29 | .43 | **.73** |
| Activities related to waste disposal^b^ | .39 | .37 | **.75** |
| Consumption-related activities^b^ | .42 | .56 | **.90** |

*Note.* Instructions preceding each item: ^a^“Please indicate the extent to which each of the following statements describes you by using the appropriate number from the scale below.^b^“In the following aspects of your daily life, how often do you think about your relationship with the natural environment.”

We then regressed engagement in proenvironmental behaviors on the mean-centered environmental identity strength and salience measures in models with the two measures as separate predictors (model 1 and model 2) and also entered simultaneously (model 3) – for overview of the statistical results, see Table A2. We found separate main effects for both, environmental identity strength and salience where salience was a stronger sole predictor. When both were entered into the model simultaneously, the strength main effect became weaker, and salience predicted engagement in proenvironmnetal behaviors more strongly. The explained variance in the data was higher with salience as the single predictor in comparison to strength as a single predictor and highest for a model with both predictors.

Table A2. Regression models (Study 1b)

|  |  |  | Dependent variable | |
| --- | --- | --- | --- | --- |
|  |  |  | *Engagement in proenvironmental behaviors* | |
| Model |  | Predictors | Coefficients | Model statistics |
| Model 1 |  | *EI strength* | *ß* = .52, *t* = 8.66, *p* < .001 | *F*(1, 198) = 75.03, *p* < .001,  _adj_R^2^ = .27 |
| Model 2 |  | *EI salience* | *ß* = .71, *t* = 14.12, *p* < .001 | *F*(1, 198) = 199.41, *p* < .001,  _adj_R^2^ = .50 |
| Model 3 |  | *EI strength* | *ß* = .22, *t* = 4.00, *p* < .001 | *F*(2, 197) = 115.28, *p* < .001,  _adj_R^2^ = .56 |
|  |  | *EI salience*^a^ | *ß* = .60, *t* = 10.63, *p* < .001 |  |

*Note. ED = Environmental Identity.* The effects are in standardized beta coefficients. ^a^VIF = 1.34.

Table B1. Description of the scales used in studies 1, 1b, 2 & 3

| Measure | Items | Response format | Cronbach’s alpha |
| --- | --- | --- | --- |
| EINS scale (Martin & Czellar, 2016) | 1. “Please choose the picture below that best describes your relationship with the natural environment.”  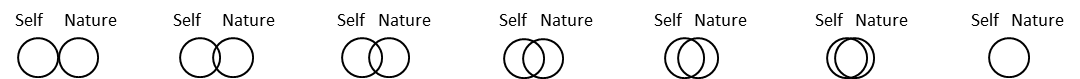  2. “Please choose the picture below that best describes nature when you think of your relationship with the natural environment.”  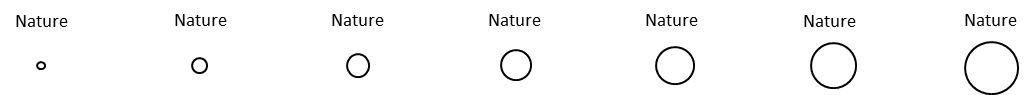  3. “Please choose the picture below that best describes your relationship with the natural environment.”  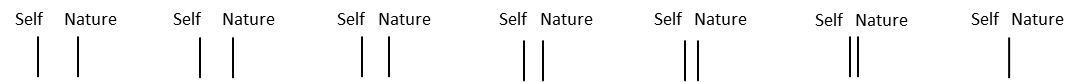  4. “Please choose the picture below that best describes your relationship with the natural environment.”  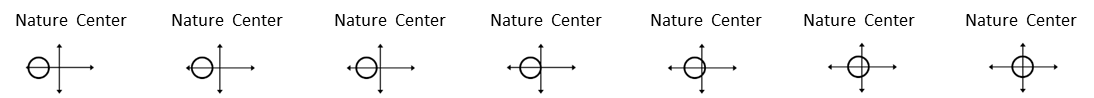 | 1 (distant) – 7 (close) graphical response options illustrating the relationship of the self with nature. | Study 1: α = .87  Study 2: α = .86  Study 3: α = .86 |
| Revised Environmental Identity scale (Clayton, et al., 2021) | “Please indicate the extent to which each of the following statements describes you by using the appropriate number from the scale below.”   1. I like to spend time outdoors in natural settings (such as woods, mountains, rivers, fields, local parks, lake or beach, or a leafy yard or garden). 2. I think of myself as a part of nature, not separate from it. 3. If I had enough resources such as time or money, I would spend some of them to protect the natural environment. 4. When I am upset or stressed, I can feel better by spending some time outdoors surrounded by nature. 5. I feel that I have a lot in common with wild animals. 6. Behaving responsibly toward nature -- living a sustainable lifestyle -- is important to who I am. 7. Learning about the natural world should be part of everyone’s upbringing. 8. If I could choose, I would prefer to live where I can have a view of the natural environment, such as trees or fields. 9. An important part of my life would be missing if I was not able to get outside and enjoy nature from time to time. 10. I think elements of the natural world are more beautiful than any work of art. 11. I feel refreshed when I spend time in nature. 12. I consider myself a steward of our natural resources. 13. I feel comfortable out in nature. 14. I enjoy encountering elements of nature, like trees or grass, even when I am in a city setting. | 1 (Not at all true of me) – 4 (Neither true nor untrue) – 7 (Completely true of me) | Study 1b: α = .93 |
| Green Consumer Self-Identity scale (Sparks & Shepherd, 1992) | 1. “I think of myself as a "green consumer." 2. “I think of myself as someone who is very concerned with "green issues." | 1 (completely disagree) – 7 (completely agree) | Study 3: α = .83 |
| Environmental identity salience in environmentally relevant domains  Self-constructed (based on environmentally relevant behavioral domains from Schultz & Kaiser, 2012) | “In the following aspects of your daily life, how often do you think about your relationship with the natural environment?”   1. House-related activities. 2. Activities related to transportation and traveling. 3. Activities related to waste disposal. 4. Consumption-related activities. | 1 (never) – 7 (very often) | Study 1: α = .80  Study 1b: α = .85  Study 2: α = .67  Study 3: α = .84 |
| Engagement in proenvironmental behaviors (Tam, 2013) | “Please evaluate how frequently you perform the following behaviors in daily life.”   1. Looking for ways to reuse things. 2. Recycling things (e.g., papers, cans, bottles). 3. Encouraging friends or family to recycle. 4. Purchasing products in reusable containers. 5. Writing a letter to public authorities to support an environmental issue. 6. Volunteering time to help an environmentalist group. 7. Buying environmentally friendly products even if they may not work as well as competing products. 8. Purchasing something made of recycled materials even though it is more expensive. 9. Buying products only from companies that have a strong record of protecting the environment. 10. Contacting public authorities to complain about environmental problems. 11. Taking a shorter shower to conserve water. 12. Using energy-efficient household devices such as light bulbs.   Extended with (in Study 3):   1. Installing an energy-efficient improvement at home, such as solar panel. 2. Traveling by public transport, biking or walking instead of using a car. 3. Living car-free. 4. Reducing your consumption of animal products. 5. Buying local products. 6. Buying goods from Swiss companies. 7. Following a plant based diet. 8. Considering to purchase an electric or a hybrid car. 9. Purchasing second hand products (e.g., clothes). 10. Refusing to buy products with excessive packaging. 11. Boycotting companies with an unecological background. 12. Not buying a product if you know the company which sells it is socially irresponsible. 13. Avoiding to buy products from a company that you know may be harming the environment. | 1 (never) – 7 (very often) | Study 1: α = .91  Study 1b: α = .91  Study 3: α = .90 |
| Donations to pro-environmental organizations | “On average, how much do you donate to support pro-environmental organizations?” | 1 – 1000 CH/year slider |  |
| Participation / Vote in a referendum about the Responsible Business Initiative | “Have you participated in the vote?”  “How did you vote?” | Yes / No/ / I am not allowed to vote in Switzerland  I voted Yes to the Initiative / I voted No to the Initiative | Study 3: α = .83 |
| Engagement in proenvironmental Christmas behaviors (self-constructed) | “In which of these behaviors do you engage / will you engage during this year's Christmas season? Please click all those that apply.”   1. Buying locally made gifts 2. Buying gifts made from recycled sources 3. Turning off tree lights and indoor/outdoor house decorative lighting at bedtime 4. Buying organic meat for Christmas dinner 5. Using eco-friendly alternatives to conventional wrapping paper 6. Reusing gift packing materials 7. Using handcrafted decorations | 0 (does not apply) / 1 (does apply) |  |

References

Clayton, S., Czellar, S., Nartova-Bochaver, S., Skibins, J. C., Salazar, G., Tseng, Y. C., ... & Monge-Rodriguez, F. S. (2021). Cross-cultural validation of a revised environmental identity scale. *Sustainability, 13*, 2387. <https://doi.org/10.3390/su13042387>

Martin, C., & Czellar, S. (2016). The extended Inclusion of Nature in Self scale. *Journal of Environmental Psychology*, 47, 181–194; https://doi.org/10.1016/j.jenvp.2016.05.006

Tam, K. P. (2013). Dispositional empathy with nature. *Journal of Environmental Psychology*, *35*, 92-104.

Schultz, P. W., & Kaiser, F. G. (2012). Promoting pro-environmental behavior. In S. D. Clayton (Ed.), The Oxford handbook of environmental and conservation psychology (pp. 556–580). Oxford University Press. https://doi.org/10.1093/oxfordhb/9780199733026.013.0029

Sparks, P., & Shepherd, R. (1992). Self-identity and the theory of planned behavior: Assessing the role of identification with “green consumerism”. *Social Psychology Quarterly*, 55, 388 - 399.
